# Death pluralism: a proposal

**DOI:** 10.1186/s13010-023-00139-3

**Published:** 2023-08-02

**Authors:** Gonzalo Díaz-Cobacho, Alberto Molina-Pérez, David Rodríguez-Arias

**Affiliations:** 1 Department of Philosophy I, University of Granada, Granada, Spain; 2 Instituto de Estudios Sociales Avanzados (IESA), CSIC, IESA-CSIC, Córdoba, Spain

**Keywords:** Pluralism, Brain death, Accommodation, Bioethics, Religion

## Abstract

The debate over the determination of death has been raging for more than fifty years. Since then, objections against the diagnosis of brain death from family members of those diagnosed as dead-have been increasing and are causing some countries to take novel steps to accommodate people’s beliefs and preferences in the determination of death. This, coupled with criticism by some academics of the brain death criterion, raises some questions about the issues surrounding the determination of death. In this paper, we discuss some of the main approaches to death determination that have been theoretically proposed or currently put into practice and propose a new approach to death determination called "weak pluralism" as a reasonable ethical and political alternative to respect diversity in death determination.

## Background

### The neurological criterion of death

In the late 1950s and early 1960s, advances in resuscitation techniques helped recover patients in increasingly dire conditions, although some of them could only survive with severe neurological sequelae. Health professionals faced an increasing number of patients with irreversible brain damage and no chance of meaningful recovery. The term that French physicians Mollaret and Goulon used to refer to this phenomenon was *coma depassé* [33], a state of irreversible unconsciousness that would be used for some years as a diagnosis for these patients.

In the late 1960s, important institutions in the field of medical science such as the French College of Physicians [1], the World Medical Association [28], and the Harvard Medical School Ad Hoc Committee on Brain Death [4] developed a new concept for the determination of a person's death: the so-called “brain death” (BD), that is, the determination of death by neurological criteria. This new concept would soon be adopted by the medical community at large –in addition to the traditional cardiorespiratory criterion– and implemented into the laws of many developed countries. Currently, at least 83 countries worldwide have protocols for the determination of brain death/death by neurological criteria [23].

BD allowed physicians to declare a patient's death before the heart stopped beating, opening up the possibility of obtaining and transplanting vital organs, including the heart itself, under optimal conditions. For this reason, BD became a fundamental element in the success of the transplant systems that every year saves and improves the lives of thousands of patients on the waiting list. In Spain, a leader country in deceased donation, more than 65% of transplants are performed with organs from BD donors [9]. In contrast, in other European countries such as Denmark, 100% of deceased organ donations come from BD patients [9].

### The brain death controversy among scholars

Although firmly established in clinical practice and accepted in many jurisdictions, the mainstream rationale for equating BD to death –that BD individuals have irreversibly lost integration– has been challenged from different perspectives [6, 7, 11, 13, 14, 16, 24, 32, 43, 50, 52, 60, 63, 65, 69, 72, 73]. Evidence shows that several integrative functions of the organism (such as the endocrine system) can continue in BD individuals [39], D. A. [54]. Moreover, BD individuals can be artificially sustained for years (D. A. [55], as shown by the case of Jahi McMath. In 2008 the President's Council on Bioethics in the U.S. rejected the “false assumptions that the brain is the ‘integrator’ of vital functions” while justifying brain death based on a new rationale [62].

In 2018, the Hastings Center published a special report in commemoration of the fiftieth anniversary of Harvard Committee’s seminal article on brain death [15]. This report shows that the debate between experts on what death is and how to determine it has never ceased and remains very active.

The sources of disagreement and controversy are many and varied, and it is beyond the scope of this article to review them all (for a brief overview of the different positions on this subject, see D. A. [56, 75]. Suffice it to say that the debates take place on metaphysical and ethical arenas, and to a lesser extent on scientific and philosophy of science arenas [30, 37].

For example, one of the philosophical issues at stake is whether human death is primarily a biological phenomenon. On the one hand, among those who believe that death is a biological phenomenon, there are differing opinions as to its nature. Although most defend the idea that death is the cessation of the functioning of the organism-as-a-whole (rather than the whole organism), there are many different interpretations of this notion. The conflicting rationales for the brain criterion of death put forward by the President's Commission [44] and the President's Council [62] are a good illustration of this, with the former defending a systemic or cybernetic conception of the organism-as-a-whole, while the latter proposed a phenomenological conception (see [49]. The source of these disputes over an issue that all parties agree is biological, i.e. scientific, is often to be found in their underlying metaphysical assumptions. However, even authors who share the idea that death is a biological phenomenon, and who also share a common underlying metaphysical position, be it materialistism, hylemorphism or other, do not necessarily agree as to whether brain death is equivalent to the death of the organism as a whole.

On the other hand, among those who dispute the claim that death is primarily a biological phenomenon, some argue that what matters is not the death of the human animal (the organism) but the loss of what constitutes a human being, or the loss of what one deems more important (morally) in human life, such as personhood or personal identity, or the soul understood in either religious or non-religious terms [25, 26, 31]. Others argue that death, the death that matters to us, is above all a social construct or a legal fiction, which are independent of the biological reality of death, if there is such a thing. These authors do not necessarily deny the biological phenomena associated with death, or that these biological phenomena are relevant in determining human death, or that biologists and physicians are capable of correctly assessing the vital biological state of a given organism. However, whether for metaphysical, ethical, legal or anthropological reasons, they consider that human death is a concept that is distinct from and irreducible to these biological phenomena. With regard to whether or not the criterion of the brain should be used to determine the death of a human being, they also have conflicting opinions.The above is only a limited overview of some of the philosophical issues that have been discussed since 1968. These discussions may make use of and draw on the scientific data, but are independent of it. For example, to our knowledge, no one would deny that hypothalamic activity, including the production of antidiuretic hormone, can remain in brain dead individuals. However, there is a debate as to whether or not this activity is functional; if so, whether this remaining brain function is relevant, significant or critical to the determination of brain death; and if so, whether these individuals are actually alive according to the law (which in the US requires the irreversible cessation of all functions of the entire brain, including the brainstem) or, on the contrary, whether the medico-legal brain criterion should be revised as to exclude hypothalamic function. In other words, the scientific data alone do not suffice to determine what death is nor science alone can end the controversies on this issue.

### The brain death controversy among health professionals and the public

The controversies associated with the concept of BD are not limited to academic forums. If we take into account the knowledge and beliefs of health professionals and the general public in relation to this concept, we observe that there is far from a consensus as to what BD is or what it implies: these assertions can be verified by surveys of health professionals and the general public in which they show ignorance or disagreement with the BD criterion [74, 75].

Some people are reluctant to accept BD as equivalent to death. A recent study in Australia showed that a substantial proportion of the general public are either unsure or do not consider that a patient described as ‘BD’ is actually dead (29,8%) [59]. Similar conclusions can be drawn from studies of the public in the USA [40, 58]. More generally, a review of the literature including 43 studies with 18,603 participants showed that “participants generally do not understand three key issues: (1) uncontested biological facts about BD, (2) the legal status of BD, and (3) that organs are procured from brain dead patients while their hearts are still beating and before their removal from ventilators.” [51].

It is not uncommon for neurologists and critical care physicians to encounter families who object to the discontinuation of life support after the declaration of death by neurological criteria [20, 64]. This opposition is illustrated by controversial cases, including Jahi McMath’s case (D. A. [55], where parents objected to the treatment withdrawal, thus initiating a legal suit [21].

Among the reasons invoked by families against BD, some are religious in nature [67]. Cultural and religious traditions have been cited to explain why several Eastern countries, including China and Japan, have been reluctant to incorporate BD into legislation and medical practice [27, 61, 70].

There may be non-religious reasons for rejecting BD among the general public, including philosophical and 'common sense' reasons, as there are among scholars. However, there is a lack of empirical data to support or refute this hypothesis. Furthermore, in those jurisdictions where it is legal to object to BD determination, to our knowledge only religious and cultural grounds are considered. Therefore, in the following we will focus on religion and culture to describe people's negative attitudes towards BD, although this may only be part of the picture.

In the USA, a recent survey found that in 20% of cases involving a religious objection, the patient was Buddhist, Hindu, Jewish, or Muslim [22]. Some evangelical Protestants, Japanese Shintoists, and Native Americans have also asserted religious objections to BD determination [42]. A survey of Jewish faith leaders found that, although 97% of rabbis know that BD and cardiopulmonary death are medically and legally equivalent, one in four believe that BD is not equivalent to death and almost one in five agrees with the continuation of life support after BD determination [19]. Opposition to BD and discontinuation of life support is particularly extended among Ultra-orthodox Jews [10]. Among Muslim faith leaders, although a majority of scholars and medical organizations accept BD as true death, the consensus is not unanimous [8, 29]. Some prominent scholars in the academic debate on BD even claim that law enforcement of a nonconsensual determination of BD violates the religious rights of observant Muslims [45].

Moreover, there are also numerous surveys in which, when health professionals are asked about the determination of death, they show ignorance or rejection of it [17, 20]. Although most health professionals involved in the care of BD individuals believe that BD is a reliable standard for death determination [47], half of them (49%), according to a survey conducted in France, the US and Spain, believe that mechanical ventilation should not be discontinued against family wishes or the formerly expressed preferences of the deceased, and even many professionals (41%) would agree that death should not be declared against their wishes [46].

### Requests for accommodation

In January 2019, the American Association of Neurology (AAN) published a position statement to provide guidance to its members “on how to respond to objections to the determination of death by neurologic criteria and requests for temporary or indefinite accommodation” [48]. The AAN acknowledges that physicians may face requests for accommodation by relatives and patient surrogates that include objections to BD determination or the withdrawal of organ-sustaining technology. These requests may originate, according to the AAN, from either a lack of understanding or actual disagreement– of brain-based determinations of death. Although the AAN strongly endorses the view that BD is death, and that there is no ethical obligation to provide medical treatment to a deceased person, it suggests that some degree of accommodation based on “reasonable and sincere social, moral, cultural, and religious considerations” may be necessary for pragmatic reasons. The AAN statement depicts its own solution as temporary and ethically unsubstantiated. In short, they play the role of a stopgap to avoid initiating legal proceedings with the families of brain-dead individuals (e.g. the case of Jahi McMath) and to avoid compromising the work and integrity of healthcare professionals.

In other contexts, however, there may be more room for negotiation and compromise based on institutional guidance, such as the aforementioned AAN statement. In the U.S., the states of New York, California, and Illinois go a step further by including accommodation clauses in their regulations or laws, the extent and duration of which are usually left to the discretion of hospitals themselves. Israel and the state of New Jersey go even further by including an option for religiously based dissent in the law, meaning that an individual who would be considered dead according to medical standards may remain legally alive if relatives veto the determination of BD on religious grounds [10, 18]. As a matter of fact, this is a form of pluralism with regard to the legal determination of death. An even stronger form of pluralism exists in Japan where the law establishes a dualistic system of eligibility in the determination of death, meaning that individuals can actually choose, in agreement with their family, by which of the two criteria (cardiorespiratory or neurological) they wish to be declared dead [3].^1^

There are several degrees of legal adaptability to religious or non-religious beliefs with regard to the determination of BD, from no accommodation at all, to the presumption that BD does not equate to death unless the individual or their family members accept that concept. The measures recommended by the AAN in the U.S. may solve a practical problem in a clinical setting where increased mistrust may be particularly harmful and counterproductive (e.g. in terms of organ procurement), but they fail to address the theoretical and political problem of whether people should have a right to object to a BD diagnosis. In fact, these forms of accommodation raise but leave unresolved the fundamental question of who has authority –and on what grounds– to draw the line between life and death.

### Some important philosophical distinctions

#### Four different levels of description

Human death can be approached from at least four different perspectives or levels of description: ontological or metaphysical, conceptual, epistemological, and legal. The order of these levels is not arbitrary, as we believe that each level can have theoretical implications for the next.

First, we must define the nature of death itself. The question is: what is death? This is an ontological/metaphysical question because it refers to reality itself, its nature and composition. A corollary question would be: when does death occur?, which is also an ontological question that refers to the causes or conditions of the transition from life to death. For example, the mainstream view is that death is a biological phenomenon that occurs when the organism ceases to function as a whole. However, the interpretation of the organism-as-a-whole concept may differ depending on the underlying metaphysical background we hold (e.g., materialism/mechanicism, Aristotelian/hylemorphism, etc.).

Secondly, there is the question of how to define death once we have reached a conclusion at the ontological/metaphysical level. For example, those who believe believe that death is a biological issue must clarify their concept of death from a biological perspective. Several authors believe that death is the loss of the *integrated functioning* of the organism as a whole, although some argue that it requires the irreversible cessation of brain function as a whole [5], some argue that it requires the irreversible cessation of systemic circulation instead, thus rejecting the brain criterion (D. A. [57], and others argue that an irreversible loss of cortical functions responsible for consciousness and cognition, which they claim are the specific capacities of the human being, would be sufficient [12]. Other concepts of death include the thesis that we die when entropy exceeds homeostasis [38]. All of them agree on the biological basis of the ontology of death, but none of them handles the same concept of biological death. Therefore, we can say that there is a *conceptual* disagreement between them.

Third, there is the question of determining whether or not death has occurred (in other words, whether or not the criteria for meeting the definition are met) and how this can be ascertained. In other words, how do we know if an individual has died? This is an epistemological question because it concerns our knowledge of the occurrence of death. Let us imagine that several experts agree on the ontological/metaphysical nature of death and on its conceptual definition. In this case, it would seem logical that they would also agree on how to diagnose death. However, there are also disagreements on this point. There are many experts who express their rejection of some tests to evaluate whether someone is dead or not. A case that exemplifies this disagreement is that of Jahi McMath. The case itself is very well explained in the report made in 2018 by the Hastings Center [15]. What interests us about this case is that some experts alluded to epistemological arguments to say that, regardless of how we understand death, this case is controversial because the encephalic death of this girl was misdiagnosed. That is, the tests were not sufficient or adequate.

Fourth, there is the issue of declaring that an individual has died. The corresponding question would be: when should we declare death? This is a legal question insofar as it concerns the consequences of death in terms of rights, duties, and responsibilities (e.g., in case of manslaughter or murder), as well as the practices (e.g., autopsy, burial, probate, insurance claims, etc.) that are permitted or prohibited depending on whether the individual is legally dead or alive.

The ontological, conceptual, epistemological, and legal approaches are independent of each other. For example, to determine whether an individual is alive or dead, something that some non-human animals are capable of doing as well [34], it is not necessary to have a prior definition, conception, or theory of what death is. This means that people can agree on the fact that someone is dead even if they have different and incompatible conceptions about the nature and meaning of death. Similarly, to declare that an individual is dead, for social and legal purposes, it is not necessary to know what death is or even to know whether that individual is really dead (however we understand by the reality of death here). Apart from cases of diagnostic and administrative errors, it is possible and lawful to declare the death of an individual despite the absence of any direct evidence, such as the finding of remains (e.g., a corpse), when that individual has been missing for a prolonged period and/or when there is good reason to believe that the person has died (e.g., a plane crash).

#### Ontological monism and pluralism

People may agree on the idea that there is a correct answer to the question “what is death?”, even if they disagree on what the correct answer is. Some believe that their own answer is correct and that the other answers are wrong. Others believe that there is an answer but that we have not yet found it. This idea that there is one and only one true answer to the question of the nature of death is what we will call ontological monism.

An alternative idea would be that the same term, "death", is used to refer to different phenomena so that the question *What is death?* can have several different correct answers. For example, one can consider from an ontological perspective that death is simultaneously a spiritual phenomenon (e.g., the separation between body and soul), a psychological phenomenon (e.g., the extinction of the capacity for consciousness, personal identity, and meaningful relationships with others), a social phenomenon (e.g., the disappearance of a citizen, i.e., a physical person with rights and duties in the public sphere, or the disappearance of a node in the network of interpersonal interactions), and a biological phenomenon (e.g., the disintegration of the organism as a whole). These responses conflict when the same individual can be considered alive or dead depending on the phenomenon under consideration. This idea that there are several simultaneously correct but not equivalent (i.e. potentially conflicting) answers to the question of the nature of death –or of the vital state of an individual– is what we call ontological pluralism.

#### Legal monism and pluralism

Since the first half of the twentieth century, several countries have required that deaths be medically certified by physicians before they are legally pronounced. However, as mentioned above, death can also be legally pronounced without any medical evidence when certain conditions are met (e.g., a plane crash). In either case, the law establishes the provisions under which death can and must be pronounced.

What we will call here legal pluralism is a situation in which there are two or more non-equivalent (i.e., potentially contradictory) sets of rules for legally pronouncing death and citizens can choose which one applies to them. To put it another way, legal pluralism in relation to death is a situation in which people can legally oppose or circumvent certain legal provisions for pronouncing death, when it concerns themselves or the death of a loved one, for religious, philosophical, or other reasons. For example, the law would allow people to decide whether or not to accept the neurological criteria for death, as determined by medical standards, for themselves and their family members. This means that, for legal purposes, the vital status of a patient with BD would depend on the decision of the patient and/or their family. In contrast, what we will call here legal monism is (most often) the situation in which people cannot make any decision about how their death will be legally pronounced because there is only one set of rules that apply to everyone equally.

#### Conceptual and epistemological monism and pluralism

If, as we have mentioned above, we share the same idea of what death is from an ontological perspective (ontological monism), we may disagree with our concept of what it means to be dead. In this case, we would be faced with conceptual pluralism. Finally, if we share the same ontological perspective (ontological monism) and also share the same conceptual perspective (conceptual monism), we could disagree on how to diagnose the death of a human being and we would be faced with epistemological pluralism. To understand these concepts in practice, it will be useful to list the main approaches that, de facto, currently exist in our societies to determine whether a person is dead or not.

## Discussion

### Approaches to the practical ways to determine death

#### Strong monism

The first position, the most common in most countries, does not dispute the precepts established by the 1981 President's Commission [44]. These precepts state that there are only two criteria for determining human death (cardiorespiratory and neurological). In fact, the legislation of most Western countries does not even question the possibility of accepting someone as alive in the case of one of these two situations. We will call this position strong monism. This approach is based on the idea that the neurological and cardiorespiratory criteria for determining death are based on objective and unquestionable scientific (biological) facts, which also have a clear ontological basis and which translate into a legal articulation in which death can only be determined in one way. Thus, we would be faced with monism on all levels: on the ontological level (since death is a biological phenomenon), on the conceptual level (because we know how to define it), on the epistemological level (since we also know how to identify it) and on the legal level (since only one way of determining death is accepted, represented by two criteria: neurological and cardio-respiratory).

#### Limits of strong monism

The strong monistic view usually takes the form of biological monism: death can only be predicated on organisms because life and death are biological phenomena. According to this view, irreversible loss of function of vital organs, such as the brain or the heart, means death. The implication is that scientists-biologists have the epistemic authority not only to confirm the loss of organ function, but also to interpret that observation in terms of life and death and to describe the event of death (i.e., the physiology of death) the fact that an organ (e.g., the brain or the heart) has irreversibly ceased to function is treated as an unobjectionable fact. According to the most frequent version of strong monism, the cessation of an organ implies death insofar as the organ (brain, heart) is necessary for the integration of the organism as a whole. When the death of a vital organ is total and irreversible, death is unquestionable. However, it is important to note that experts who agree on the idea that death is a biological phenomenon and who share the definition of death as a loss of integration may still disagree on the vital status of those individuals. Thus, disagreements within the strong biological monism position may stem from: (a) the belief that standard tests for assessing loss of vital organ function are unreliable; (b) the belief that the organ in question –e.g., the brain– is not essential for integration, meaning that its functional loss would not necessarily imply death (biologically understood as a loss of integration).

But there are not only conceptual or epistemological disagreements about the validity of the monism position, there are also ontological disagreements. This type of disagreement differs from the strong monism position in questions related to the nature of the phenomenon and can be divided mainly into two categories: (a) First, there are those who argue that death is not a strictly biological phenomenon, but rather a hybrid phenomenon, with one foot in biology and the other in culture; (b) second, there are those who argue that death is a social construct and that therefore science has no special relevance for decision-making related to it. Within this idea of social construction, insofar as death is not something biological, we can find different currents: some defend that the social construction of death refers to religion, others say that it refers to culture while others say that death responds to philosophical postulates. In this last place, we find those who defend that we cannot define death without speaking of metaphysics.^2^

#### Weak monism

The second position, weak monism, shares with the strong monism approach the idea that there is only one correct way to interpret the vital status of individuals with BD as dead and only one ontological basis for determining death. However, this view adds a clause whereby patients or their relatives can reject declarations of death (in BD diagnoses) for pragmatic reasons (e.g., to avoid lawsuits or to preserve trust in healthcare professionals). In practice, such a clause allows individuals to maintain a living state even though anyone else in the same conditions would be considered dead. Although this approach allows the wishes of families to be respected, it presupposes that they lack valid reasons for disagreeing with the declaration of the death of their family member. In essence, the accommodation stance is nothing more than a stopgap to silence the voices of those who disagree with the neurological criteria for the determination of death, without questioning the soundness of their objections to the real underlying problem.

An example of this type of posture is found in many states in the United States, where for practical reasons and out of respect for the religious beliefs of many people, certain people are allowed to be accommodated. That is, they are treated as alive despite being in BD status. We could say that this type of monism is framed in the same ontological, conceptual and epistemic level as strong monism but that it would broaden its framework of options in the legal plane to accommodate these accommodations.

#### Limits of weak monism

Weak monism differs in practice from strong monism in that it tolerates objections to the determination of BD by allowing accommodation procedures. However, both forms of monism reject that the reasons for such objections can be acceptable, since they are based exclusively on extra-biological, non-scientific grounds. In other words, although weak monism is a practical mechanism for dealing with religious and cultural disagreements, it shares with strong monism the fundamental assumption that disagreementsabout the determination of death have no epistemic credibility.

Both forms of monism also face the objection that, while physicians certainly have the expert authority to describe the state of a body part such as the brain, they do not have the similar ability to demonstrate that the brain is vital. To claim so implies the imposition of a biological definition of death.

But not only are there conceptual disagreements about the validity of the position of monism, there are also ontological disagreements. Weak monism falls into the same error as strong monism in that it accepts that the reasons for a patient to make use of these accommodations lie exclusively in non-scientific matters. In other words, although it responds to religious and cultural disagreements, it makes the same mistake as one of the positions of strong monism: it does not consider conceptual disagreements.

#### Strong pluralism

Strong pluralism allows individuals to choose whether they want to be considered alive or dead, regardless of scientific issues. In Japan, for example, the law provides for a pluralistic system of eligibility in the determination of death whereby individuals or their relatives can choose by which of two criteria (cardiorespiratory or neurological) they wish to be declared dead.

Neither strong nor weak monism questions the conceptual validity of the BD criteria; they simply equate such conceptual validity with other values such as religious or cultural values, giving them sufficient weight so that the neurological criterion can be rejected on the basis of such values.

What this kind of pluralism adds to simple accommodation is that death is conceived, not as a natural kind to be discovered by simple observation, but as a socially constructed phenomenon about which clinicians have no particular expertise and should not have the last word. Japan and New Jersey are territories where this approach has been discussed [2, 36, 41]. In both, the response of the authorities has been to seek a position that safeguards, on the one hand, the criteria for determining death proposed by the medical community and so far, accepted in almost all countries, and on the other, the voices of those who do not share those criteria. This task has been accomplished through the application of a dualistic model in the determination of death, which allows individuals to decide, in cases of complete BD, whether they should be considered alive or dead. By situating strong pluralism at the different levels, we have used above, we could say that this type of pluralism is situated in an ontological pluralism (insofar as neither of the two conceptions predominates) and can be situated in a conceptual and epistemological pluralism or monism. Finally, at the legal level, it would also be situated in pluralism. It is thus a form of ontological and legal pluralism.

#### Limits of strong pluralism

Strong Pluralism can be criticized, fundamentally, from two perspectives. First, it can be criticized for its lack of conceptual rigor. If we approach the determination of death from a perspective in which the weight we give to empirical evidence is high, Strong Pluralism lacks arguments to support it since its principles are based on law and ontology. We could question this type of pluralism from the perspective that moral, cultural and religious reasons should not have equal weight in the determination of death. However, this criticism could presuppose a false dichotomy between science and cultural or religious ideas, when in fact the ontological (metaphysical) question is inevitably implicated in the debate about the determination of death. Whether implicitly or explicitly, all sides operate with ontological assumptions, and it is unfair to describe all those who disagree with the scientific consensus (which of course has an ontological presupposition at its base) as operating from "cultural or religious" assumptions. Certainly, an objection against Strong Monism that justifies a Strong Pluralism (i.e., alien or at least not justified by scientific issues) may originate in a different metaphysics that is itself compatible with the scientific data used by the proponents of Strong Monism.

The second major problem with this type of pluralism lies in the difficulty of imposing legal limits on a question of an ontological nature. Unlike the weak pluralism that we will defend below, it is much more complex to limit the concept of death based on social, cultural or religious issues alone than the concept of death based on primarily biological issues.

### Our proposal

So far, we have tried to describe in a structured way the different levels that exist to describe the phenomenon of death (Ontological, Conceptual, Epistemological and Legal). This structure has also helped us to categorize the ways of determining death in practice (Strong Monism, Weak Monism and Strong Pluralism). We have also used this categorization to mention some of the objections or problems that each of these practical approaches may have.

Our main objective in this paper is to outline a practical alternative to those mentioned above that is consistent with the levels of description and allows us to reconcile the positions of those who disagree on what is considered death. To do so, we must first clarify what we mean by death.

Some authors have argued that death is a social, cultural or religious phenomenon [3, 66] and that it should be society that agrees when we can define a person as alive or dead. This would be a proposal that could be framed within Strong Pluralism from different perspectives –legal, ontological, conceptual and even social–. Our conception of death, however, does not start from this premise, and in fact, we believe that it is a mistake not to grant biology the authority it deserves. On the other hand, a proposal of this magnitude presents a practical difficulty (almost impossibility) in establishing a (limited) set of definitions of death based solely on social considerations.

Other authors, as we have already mentioned above, consider death to be strictly a biological phenomenon (they defend an Ontological Monism) and whether or not they believe that there is a unique biological concept of what death is, they do defend that we should not take into account, in any case, extra-biological values to determine what it is to be dead.

In our case, we understand death as the irreversible cessation of the vital functions of an individual. A phenomenon that has a strong biological foundation but is also marked by a series of issues that go beyond biology (beliefs, culture, society). We could say that death has a big foot in biology and a small foot in culture [71].

If we start from our definition of what it means to be dead, the solution to end the controversy in the determination of death will necessarily involve the search for a normative consensus on what we understand death to mean as a society under a biological framework. As we have shown in the section on controversies, many bioethicists have criticized, from different approaches, that BD is comparable to human death. On the contrary, those who believe that BD is comparable to human death consider that this phenomenon is solidly established. Some of these experts have proposed other alternatives to clarify this criterion of BD or to change it to another criterion. Alan Shewmon listed some of the main alternatives for the determination of BD (D. A. [56].^3^

Although these proposals are of great interest, we believe that they are solutions that do not take into account all aspects of this problem. Our proposal, however, does not aim to solve the problem per se (since for this it is necessary to go through a previous debate and seek a collective consensus), but aims to allow well-founded disagreementsnot to be disregarded while this controversy lasts. To this end, we believe that articulating a Weak Pluralism is the best theoretical and practical solution.

Weak Pluralism bases its rationale on the following reasoning: there is disagreement among experts as to which biological concept is the best for determining death, and furthermore, the choice of what it means to be dead involves not only factual issues. Because reaching consensus on which normatively mediated biological definitions are most appropriate is complex, we must allow people who have legitimate disagreements with the current model for determining death to object to being treated under that parameter as long as the controversy remains open.

To accomplish this, we propose to use Weak Pluralism: a concept that gives practical ability to these people to choose the criteria (cardiorespiratory or neurological) by which they want to be considered dead. Thus, a person who believes the way they can be determined dead is not really death –since they base their reasoning on the idea that the brain is not necessarily the integrating organ of our body– could be considered alive until they suffer a cardiorespiratory death.

At first glance, this proposal does not seem to be very different from Strong Pluralism or Weak Monism. However, the main difference lies not in how Weak Pluralism would be applied, but in its rationale and justification. As we have pointed out above, the justification of Weak Pluralism arises from the need to give a global answer to a problem at the ontological, conceptual, epistemic and legal level of our initial scheme. People should have the right to decide by which criteria they want to be declared dead because, in fact, there is no expert consensus on whether a person in a state of full BD is dead or not.

If these disagreements as to when a person is dead or not are resolved (in a collective deliberation mediated by biology), we believe that the definition that has been agreed upon by default should be used, allowing people who still disagree with that decision to avail themselves of a sort of Weak Monism in which they can be treated as alive even if, de facto, society does not consider them as such. The limits of these accommodations would be mediated, again, by biology and by the range of disagreementthat experts consider reasonable (generally, not accepting BD and being diagnosed in cardiorespiratory death).

### Limits, feasibility, and risk of pluralism?

Because of its ethical, legal, conceptual, and biological implications, we are aware that this proposal cannot escape some disputes, complex approaches, and uncomfortable questions. Far from dodging them, we believe that it is intellectually mature to address possible objections to our proposal and try to resolve them or at least dismantle them as far as possible.

Would it be acceptable for us to consider a body in an advanced and generalized state of decomposition as alive, just because that person, a long time ago, said that they should be treated as alive? Or conversely, would it be acceptable to consider an apparently healthy person, in their youth, to be dead just because they considered that their existence was not a form of being alive? Would it be acceptable for parents to consider their newborn baby to be dead? Are the limits set by the Harvard Ad Hoc Committee for the determination of BD equally valid for a deeply religious country (such as Israel or Iran) as for a country where secularism is firmly rooted (such as France)? Do Japan, or the state of New Jersey, entail an anomaly by accepting the disagreement in the diagnosis of BD? Is there a problem in doing so? These are some of the questions that could be asked in relation to what the limits of pluralism are. Certainly, it is a problematic question for this proposal to which we must seek an answer.

One of the biggest problems in setting limits to pluralism lies in deciding how far we think it is sensible to extend the margins. While it is true, as mentioned above, that there are de facto countries that have bordered on at least one of the two criteria for determining BD, it is also true that in no case has it been decided to go further to broaden the criteria, for example, to include persons who are in a state of higher BD.

The “higher brain” standard of death defines death as the irreversible loss of function of the higher brain, which involves the permanent incapacity to return to consciousness (as opposed to a temporary incapacity to return to consciousness, for example during sleep). No jurisdiction has adopted the “higher brain” standard, but several scholars have defended it as the best way of reconceptualising death in modern medicine […] Proponents of the “higher brain” standard of death commonly argue that the essence of human life lies in being a person with some basic awareness or understanding of the self. On this view, death occurs when personhood is permanently lost [35].

A possible solution to this problem was formulated by Robert Veatch and Laine Ross in their book *Defining Death: The Case for Choice* in which they advocate agreeing on a range of reasonable positions within what is plausible. That are the three positions regarding the determination of death mentioned here, which, although they generate dissent among experts, are the only ones considered debatable (cardiorespiratory death, BD, and higher brain death) [68].

The argument employed by Veatch and Ross is precisely the one that would prevent our argument from falling down a slippery slope: from a conceptual perspective, we do not have the knowledge necessary to establish when a person is dead according to certain criteria. However, we do have the ability to determine which of those criteria are the ones that are the margins of pluralism (by excess: cardiorespiratory criteria, by default: higher brain death).

Finally, it is important to note that the fact that a person can choose one or the other definition of death does not imply that they must in fact choose it, only that if they wanted to choose it, they could do so. That is, regardless of the limits set by experts, no one can force another person to choose a certain criterion of death, they can only offer the choice.

Other common criticisms of pluralism are related to its unfeasibility. However, in this article two key issues have been put forward to defend pluralism against this criticism: on the one hand, it has been shown that, in certain communities, de facto a legal pluralistic system already exists, which invalidates theses related to the chaos that can be generated by extracting this debate from the purely medical sphere (Japan and New Jersey). On the other hand, the mere fact that the theory is formulated in legal terms implies that in a democratic country there should not be insurmountable obstacles to deliberation and subsequent implementation of the decisions taken in that deliberation.

The way to corroborate that a person wants to be treated differently in the determination of their death can be easily reflected through their own advanced living will or through their relatives. As in some cases with organ donation, the procedure for finding out whether someone wanted to be treated in an exceptional way should not make the situation more complex.

Problems arising from a change in a person's life status could indeed be aggravated in the case of a pluralistic system. Thus, we could think that a self-interested and malicious son (or perhaps a son who is neglected by society and in dire social circumstances) would bet on keeping his father linked to a series of artifices by considering him legally alive in order to collect his pension for a longer period of time. We could also think of the opposite case, where this same son decides, while firmly believing that his father is alive, to proceed to disconnect him in order to receive a luxurious house in inheritance.

For these reasons, some authors have been careful to protect themselves from such criticisms in relation to pluralism (or similar alternatives) in death by trying to counter such accusations with arguments that show that, while some might try to take advantage of the loopholes of a possible implementation of a pluralistic system, there would be no substantive differences with the problems that exist today in relation to those who are diagnosed in an irreversible coma or minimally conscious state [68].

Just as we assume that there may be abuses in matters such as euthanasia or therapeutic obstinacy, we must also assume that there may be abuses if we were to apply a pluralistic model. While it is true that society can impose mechanisms to prevent these abuses, we could never completely eliminate them, only fight vigorously to try to keep them to a minimum.

## Conclusions

In this paper we have tried to offer a novel response to a problem that is becoming a classic in the bioethics literature and that continues to cause rivers of ink to flow among academics, health professionals and all those involved in general.

Our objectives at the beginning of this work were twofold: on the one hand, we wanted to conceptualize the different levels of discussion on this issue –ontological, conceptual, epistemic and legal–. On the other hand, we have proposed to outline what could be a practical solution to a theoretical problem that is difficult to solve.

Certainly, we believe that finding a consensus or a solution to this debate is an arduous and incredibly complex task. However, we believe that it is imperative to provide a refuge for all those people who have legitimate and reasonable disagreements with the way in which human death is currently determined. To this end, we have designed this proposal which we hope will serve to resolve the practical problems arising from the theoretical controversy, allowing all parties to feel satisfied during the time it takes to find a reasonable and reasoned solution.

## Data Availability

No data, materials and/or code available.

## Abbreviation

BD: Brain Death

## Footnotes

1 At this moment, objections to brain death are not accepted on philosophical grounds. There are different ways of approaching death from the metaphysical perspective and in some of them, for example Aristotelian or Thomistic metaphysics, it is not entirely clear whether brain death is acceptable.

2 Some authors have emphasized the importance of metaphysics in the determination of death and the. concept of brain death. See Josef Seifert and Alan Shewmon [50], A. [53].

3 In this article, Alan Shewmon proposes four alternatives to the "bifurcated structure of the 1981 UDDA": *1. The biological concept of death, 2. A non-biological, consciousness-based, "higher brain" concept of death, 3. The physiological-psychological hybrid of irreversible apneic unconsciousness and, 4. Recognize BD explicitly as a legal fiction or legal status*. All these alternatives are collected and explained in the article Statement in Support of Revising the Uniform Determination of Death Act and in Opposition to a Proposed Revision (D. A. [56].
